# Examining the Relationship Between Parent and Child Psychopathology in Treatment-Seeking Veterans

**DOI:** 10.1007/s10578-017-0743-y

**Published:** 2017-06-28

**Authors:** Alyson K. Zalta, Eric Bui, Niranjan S. Karnik, Philip Held, Lauren M. Laifer, Julia C. Sager, Denise Zou, Paula K. Rauch, Naomi M. Simon, Mark H. Pollack, Bonnie Ohye

**Affiliations:** 1 Road Home Program, Department of Psychiatry, Rush University Medical Center, 1645 W. Jackson Blvd, Suite 602, Chicago, IL 60612 USA; 2 Department of Behavioral Sciences, Rush University Medical Center, Chicago, IL USA; 3 Home Base, a Red Sox Foundation and Massachusetts General Hospital program, Boston, MA USA; 4 Department of Psychiatry, Harvard Medical School, Boston, MA USA

**Keywords:** Veteran, Military, Parenting, Child psychopathology, Parenting sense of competence

## Abstract

This study aimed to examine: (1) the relationship between parental psychopathology and child psychopathology in military families and (2) parenting sense of competence as a mediator of the relationship between veteran psychopathology and child psychopathology. As part of their standard clinical evaluations, 215 treatment-seeking veterans who reported having a child between the ages of 4 and 17 were assessed for psychopathology (posttraumatic stress disorder, depression, anxiety, and stress), their sense of competence as a parent, and their child’s psychopathology (internalizing, externalizing, and attentional symptoms). A path analysis model examining parenting sense of competence as a mediator of the relationship between veteran psychopathology and child psychopathology showed significant indirect effects of veteran depression on all child psychopathology outcomes via parenting sense of competence. Parental sense of competence may be a critical mechanism linking veteran depression and child psychopathology, and may therefore be an important target for intervention.

## Introduction

Since September 11, 2001, over 2.6 million US service members have deployed to Iraq and/or Afghanistan, and this number is projected to reach 3.5 million by 2019 [1]. Operation Enduring Freedom, Operation Iraqi Freedom, and Operation New Dawn are fundamentally unique from previous wars and are characterized by repeated and extended deployments, with early evidence suggesting disproportionately high rates of mental health disorders compared to the general US population [2]. The post-9/11 conflicts tax not only military personnel, but also their immediate family members, including 2 million military-connected children, hundreds of thousands of whom have experienced at least one parental deployment [3]. Both deployment and reintegration are accompanied by unique stressors for military families, which may be particularly salient for dependent children. These include renegotiation of family roles, responsibilities, and boundaries; changes in routines; lack of awareness about military service within communities; and media exposure that may present incomplete information or negative aspects of deployment [4–6].

While children generally adapt well to mild and short-lived stressors [7], sustained severe stress may result in significant behavioral and emotional disturbances, especially when support is not readily available [4]. It has been suggested that up to a third of military-connected children are at risk for decrements in emotional, cognitive, or physical functioning during parental deployment [8]. Two recent publications reviewed the literature on behavioral and emotional difficulties of military children during or after parental deployment [9, 10]. White and colleagues reviewed nine studies (sample sizes ranging from *n* = 57 to *n* = 642,397) including two longitudinal studies and found that parental deployment was associated with increased emotional and behavioral difficulties in children [9]. Card et al. conducted a meta-analysis of 16 studies (pooled *n* = 19,172), including three longitudinal studies, confirming this association [10]. Moreover, greater overall maladjustment, increased internalizing and externalizing problems, and poorer academic performance were the strongest during middle childhood, suggesting that children aged 6–12 years old may need more support during and after parental deployment relative to younger children and adolescents.

Prior research suggests that parental psychopathology across a range of conditions may be a risk factor for child psychopathology [11]. This suggests that elevated emotional and behavioral problems in military-connected children might stem from parental deployment-related psychopathology, given the high rates of mental health conditions such as depression and posttraumatic stress disorder (PTSD) in returning veterans. Moreover, there have been several studies examining how trauma exposure in military parents may contribute to child risk for emotional and behavioral problems [12]. For example, in a cross-sectional study of 272 military children, Lester et al. reported that parental PTSD symptoms were associated with child depression, as well as child internalizing and externalizing behaviors [13]. In this study, child internalizing behaviors were also strongly associated with parental depression and anxiety symptoms. These preliminary results need further replication and were limited by the restricted range of parental symptoms, as participants were not specifically treatment seeking.

It has been hypothesized that children are indirectly affected by the military by way of parental stress and psychopathology, with parenting factors mediating this relationship [14]. One potentially important parental factor is parenting self-efficacy or sense of competence. Prior research, predominantly cross-sectional, suggests that poor parenting self-efficacy is negatively associated with child adjustment and is positively associated with depressive symptoms and anxiety symptoms in veteran parents [15, 16]. Thus sense of competence in parenting might be involved in the association between veteran symptoms and psychopathology in their children. However, to date, this hypothesis has not been tested. The present study aimed to examine: (1) the relationship between veteran and child psychopathology; and (2) parenting sense of competence as a mediator of the relationship between veteran psychopathology and child psychopathology.

## Method

### Participants and Procedures

Data for the present study were collected as part of standard clinical intake evaluations conducted at one of two community, non-VA outpatient mental health clinics treating veterans and service members: Home Base, a partnership between the Red Sox Foundation and Massachusetts General Hospital (MGH; Boston, MA) (*n* = 176) and the Road Home Program at Rush University Medical Center (Chicago, IL) (*n* = 39). Both outpatient clinics offer mental health services to veterans, service members, and their families free of charge. The combined study sample is comprised of 215 veterans and service members who reported having at least one child between the ages of 4 and 17 years and completed the Pediatric Symptom Checklist-17 (PSC-17) [17]. Patients in this sample had a mean age of 37.4 years (*SD* = 7.9) and 13.3% (*n* = 28) were female. Additional demographic characteristics are displayed in Table 1.

**Table 1:** Demographic characteristics

Characteristic	*n* (%)
Male (*n* = 211)	183 (86.7)
Married, engaged, or partnered (*n* = 204)	123 (60.3)
Deployed (*n* = 176)	143 (81.3)
Military rank (*n* = 190)	
Junior-level enlisted	57 (30.0)
Non-commissioned officer	108 (56.8)
Officer	25 (13.2)
Military branch (*n* = 205)	
Army/Army Reserve	83 (40.5)
Marines/Marine Reserve	41 (20.0)
Navy/Navy Reserve	18 (8.8)
Air Force/Air Force Reserve	12 (5.9)
National Guard	50 (24.4)
Coast Guard	1 (0.5)
Military status (*n* = 204)	
Active Duty, Reserves, Inactive Ready Reserve, National Guard	77 (37.8)
Discharged, Retired, Medically Retired	127 (62.3)

### Measures

#### Veteran Demographic Information

Veterans’ demographic information including age, sex, relationship status, whether or not they deployed while in the military, military rank, military branch, and military status were gathered during the intake evaluation process.

#### Veteran Measures

##### Depression, Anxiety, and Stress

Symptoms common to depression and anxiety were assessed using the depression anxiety stress scales (DASS-21) a 21-item self-report measure with three subscales for the respective symptom clusters [18]. Veterans were asked to rate how much each statement applied to them over the past week on a 4-point Likert scale, ranging from 0 (*Never*) to 3 (*Almost always*). Scale scores are calculated by summing the seven items in each scale such that higher ratings represent higher levels of pathology. Studies indicate that the depression (DASS-D), anxiety (DASS-A), and stress (DASS-S) scales are able to distinguish well between the features of dysphoria, acute physiological arousal, and chronic tension, respectively [19]. Studies with a variety of samples, including veterans, have demonstrated that the DASS-21 has good validity and reliability [20, 21]. Internal consistency reliability for the depression, anxiety, and stress subscales of the DASS-21 in the present study were 0.92, 0.89, and.90, respectively.

##### Posttraumatic Stress Disorder

Home Base assessed PTSD using the PTSD Checklist—Civilian Version (PCL-C) a 17-item self-report measure assessing symptoms PTSD based on the diagnostic and statistical manual (DSM) Fourth Edition [22]. Veterans were asked to report “how much each problem has bothered them during the past week” on a 5-point Likert scale, ranging from 1 (*Not at all*) to 5 (*Extremely)*. The PCL-C has been shown to have good validity and reliability in veteran samples [23, 24]. The Road Home Program assessed PTSD using the 20-item self-reported PTSD Checklist for DSM-5 (PCL-5) [25]. Veterans were asked to report “how much they have been bothered by the worst event in the last month” on a 5-point Likert scale, ranging from 0 (*Not at all*) to 4 (*Extremely)*. Similar to the PCL-C, the PCL-5 has been validated and shown to have good reliability in samples of veterans [26, 27]. In order to be able to combine the two samples for the present study, the 16 common items on the PCL-C and PCL-5 were used to calculate a total score. Internal consistency for the modified version of the PCL used in this study was 0.96, indicating good reliability of this modified measure.

##### Parenting Sense of Competence

Veterans’ sense of parental competence was assessed using the Parenting Sense of Competence Scale (PSOC) [28], a 16-item self-report assessment. Veterans are asked to report the extent to which they agree with various parenting-related statements on a 6-point Likert scale ranging from 1 (*Strongly disagree*) to 6 (*Strongly agree*). Higher scores reflect a greater sense of competence in one’s parenting. The PSOC has been shown to have good validity and reliability in a variety of samples, including veterans [15]. Internal consistency for the present study was 0.86.

#### Child Measures Completed by the Veteran

##### Pediatric Symptoms

The Pediatric Symptom Checklist-17 (PSC-17) was used to assess mental health symptoms in veterans’ offspring [17]. The PSC-17 is a 17-item self-report screening tool completed by children’s caregivers. The PSC-17 has been found to have good validity and reliability and to provide comparable results to other well-established pediatric symptom measures [29–32]. The PSC-17 contains three subscales that capture internalizing (PSC-I), externalizing (PSC-E), and attention symptoms (PSC-A). Internalizing, externalizing, attention problems, and total symptoms are present at clinically significant levels if subscale scores are greater than or equal to 5, 7, 7, and 15, respectively [17]. At Home Base, veterans were asked to complete the measure for the child about whom they were most concerned. At the Road Home Program, veterans were asked to complete the PSC-17 on all of their children; to combine the Road Home data with the Home Base data, the most symptomatic child was identified and selected for analysis based on the highest total PSC-17 score. Internal consistency for the internalizing, externalizing, and attention subscales for the present study were 0.82, 0.86, 0.83, respectively. Internal consistency for the total score was 0.91.

### Procedures

As part of their initial clinical evaluation, patients with a child between the ages of 4 and 17 completed self-report screening measures including the DASS-21, PCL-5, PSOC, and the PSC-17. Demographic and diagnostic data were routinely collected as part of the baseline clinical assessment of each patient. Clinical data were maintained in two database repositories approved by the Massachusetts General Hospital (Partners Healthcare) and the Rush University Medical Center Institutional Review Boards with a waiver of consent because all assessments were collected as part of routine clinical care procedures.

### Data Analyses

Independent samples *t*-tests were conducted to test for site (MGH vs. Rush) differences in veteran psychopathology, offspring psychopathology, and parenting sense of competence. There were no significant differences on these measures by site (all *p* > .24). Given the similar intake evaluation processes at Home Base and the Road Home Program and the lack of statistically significant differences in any of the independent variables, mediators, or dependent variables, we combined the sample. We then conducted preliminary analyses to evaluate the relationship between possible demographic covariates and PSC-17 scores. Men reported that their child experienced lower symptoms on the PSC-17 internalizing subscale than women did; thus, parent sex was included as a covariate in subsequent mediation analyses involving the internalizing subscale. Veteran age, relationship status, deployment status, military rank, military branch, and military status were not significantly associated with PSC-17 scores. Therefore, they were not included as covariates in mediation analyses.

Out of the 215 participants, 32 were missing scores on the modified PCL, DASS-21, or PSOC. The presence of missing data was unrelated to any of the PSC-17 variables, suggesting that the data were missing at random (MAR). Studies have shown that full-information maximum likelihood estimation (FIML) is superior to pairwise or listwise deletion under conditions of MAR [33]. Thus, we conducted FIML using Mplus version 7.3 [34] to run all analyses. Based on results from bivariate analyses, we thus tested a path analytic model examining PSOC as a mediator of the relationship between veteran psychopathology and child psychopathology. In order to determine model fit, relative *Χ* ^*2*^, root mean square error of approximation (RMSEA), comparative fit index (CFI), and standardized root mean squared residual (SRMR) were assessed. A *Χ* ^*2*^/*df* below 3.0, an RMSEA below 0.06, a SRMR below 0.08, and a CFI above 0.9 were considered to denote adequately-fitting models [35–37]. In addition, we calculated the 90% confidence interval (CI) for the RMSEA, and the p-value for test of close fit RMSEA ≤ 0.05.

## Results

### Rates of Psychopathology in Children

Based on established cutoffs for the PSC-17, 15.3% of veterans reported having a child with significant internalizing pathology, 14.0% of veterans reported having a child with significant externalizing pathology, 18.1% of veterans reported having a child with significant attentional problems, and 30.2% of veterans reported that they had a child that met clinical threshold for at least one of the three subscales (internalizing, externalizing, attention). Based on the established cutoff for the PSC-17 total score, 20.5% of veterans reported having a child with significant overall psychopathology.

### Bivariate Analyses

The estimated correlation matrix examining the relationships between veteran psychopathology, parenting sense of competence, and child psychopathology is reported in Table 2. Veterans with higher scores on the DASS-21 depression and anxiety scales reported higher rates of internalizing symptoms in their children. Veterans with higher scores on the DASS-21 stress scale reported higher attentional symptoms in their children. Overall, the magnitude of these relationships was small to moderate. PTSD symptoms in veterans were not associated with child psychopathology.

**Table 2:** Estimated correlation matrix of veteran psychopathology, parenting sense of competence, and child psychopathology

	PSC-I	PSC-E	PSC-A	PSOC
PSOC	−0.380***	−0.391***	−0.297***	
Modified PCL	0.055	−0.005	0.118	−0.105
DASS-D	0.200**	0.062	0.126	−0.276***
DASS-A	0.135*	0.022	0.119	−0.218**
DASS-S	0.130	0.053	0.208**	−0.170*

*N* = 215. Estimated correlation matrix based full information maximum likelihood imputation in Mplus version 7.3

*PSOC* parenting sense of competence, *Modified PCL* combined PTSD Checklist score, *DASS-D* DASS-21 depression scale, *DASS-A* DASS-21 anxiety scale, *DASS-S* DASS-21 stress scale, *PSC-I* Pediatric Symptom Checklist-internalizing scale, *PSC-E* Pediatric Symptom Checklist-externalizing scale, *PSC-A* Pediatric Symptom Checklist-attention scale

*p < .05, **p < .01, ***p < .001

There was a strong negative relationship between parenting sense of competence and all child psychopathology subscales (*r* from −0.294 to −0.420, all *p* < .001). Higher scores on parenting sense of competence were also associated with lower scores on the DASS-21 depression, anxiety, and stress scales (*r* from −0.170 to −0.276, all *p* < .05). Parenting sense of competence was not associated with veteran PTSD symptoms (*r* = −0.105, *p* = .125).

### Mediation Model

**Fig. 1:**
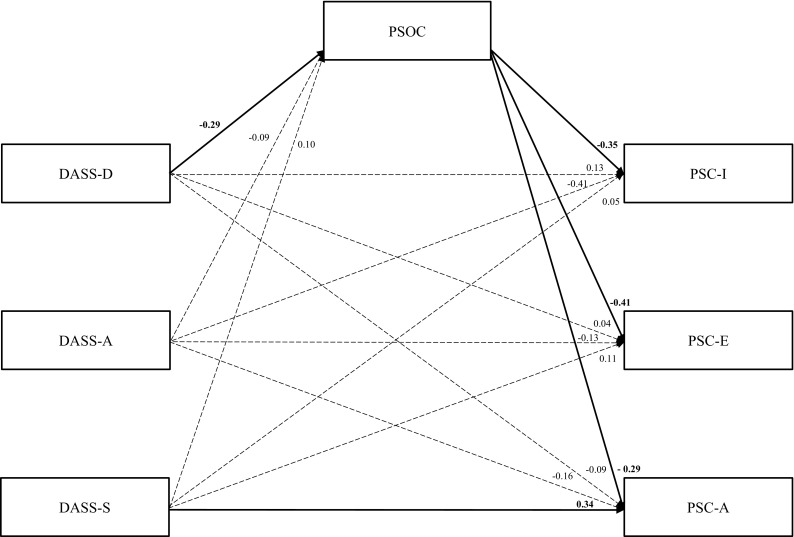
Path analysis model examining parenting sense of competence as a mediator of the relationship between veteran and child psychopathology (χ^2^(1) = 0.384, p = .54; CFI = 1.00; RMSEA = 0.000, *p* = .63; SRMR = 0.009)

Based on the bivariate analyses, we conducted a path analysis examining parenting sense of competence (PSOC) as a mediator of the relationship between veteran psychopathology (DASS-D, DASS-A, DASS-S) and child psychopathology (PSC-I, PSC-E, PSC-A). PTSD symptoms were not included in the model because they did not correlate with the PSOC. The structural model included direct paths from veteran psychopathology to child psychopathology as well as indirect paths via parenting sense of competence. Sex was included as a covariate by including direct paths from sex to all outcome variables. All exogenous variables were correlated with each other. We used full information maximum likelihood imputation and the MLR estimator, which is robust to non-normality. The model is depicted in Fig. 1 along with standardized path coefficients.

**Fig. 2:**
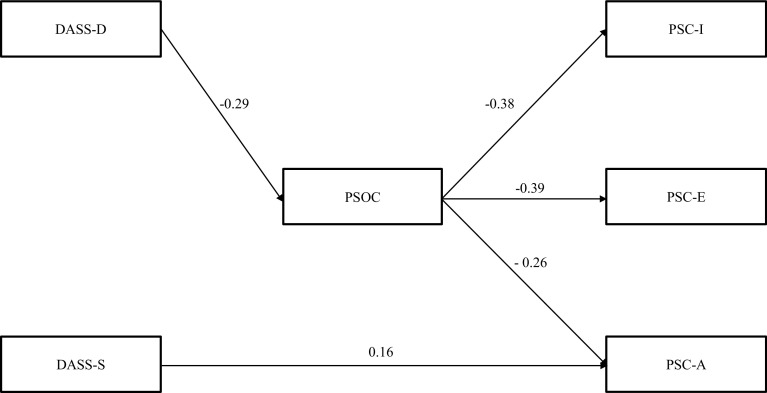
Reduced path analysis model examining parenting sense of competence as a mediator of the relationship between veteran and child psychopathology (χ^2^(8) = 8.840, p = .36; CFI = 0.996; RMSEA = 0.022, *p* = .69; SRMR = 0.029)

The overall model showed adequate fit to the data (χ^2^(1) = 0.384, p = .54; CFI = 1.00; RMSEA = 0.000, *p* = .63; SRMR = 0.009). Results showed significant indirect effects of depression (DASS-D) on all three PSC-17 outcomes via PSOC (PSC-I: Estimate = 0.019, SE = 0.009, *p* = .028; PSC-E: Estimate = 0.032, SE = 0.013, *p* = .015; PSC-A: Estimate = 0.020, SE = 0.009, *p* = .027). There was also a significant direct effect of stress (DASS-S) on child attention problems (PSC-A). This model explained 14–18% of the variance in the outcome variables. We re-ran the model without the insignificant paths to determine whether the model continued to have adequate fit once the model was less saturated (Fig. 2). The reduced model also demonstrated adequate fit to the data (χ^2^(8) = 8.840, p = .36; CFI = 0.996; RMSEA = 0.022, *p* = .69; SRMR = 0.029). All direct paths remained significant and all three indirect paths from depression (DASS-D) to the PSC-17 outcomes via PSOC remained significant (PSC-I: Estimate = 0.021, SE = 0.008, *p* = .007; PSC-E: Estimate = 0.030, SE = 0.009, *p* = .001; PSC-A: Estimate = 0.017, SE = 0.007, *p* = .008). This model explained 12–17% of the variance in the outcome variables.

## Discussion

Rates of child psychopathology were high in our sample of treatment-seeking veterans and service members. We found almost twice the rate of children screening positive for overall psychopathology compared to a national outpatient pediatric sample (20.5% met the cutoff for the PSC-17 total score in current sample versus 12% in national sample) [32]. These findings suggest that screening children of veterans and service members may be indicated as this would enable early identification of psychopathology in this high-risk sample. In particular, our findings suggest that veterans with higher rates of depression and anxious arousal may be more likely to have children with higher rates of internalizing symptoms. Additionally, veterans with higher rates of chronic tension may be more likely to have children with greater attentional problems. Thus, targeted screening of children may be particularly warranted in families where veterans are experiencing symptoms of depression and anxiety. Of note, the effect size of the relationships between veteran psychopathology and child psychopathology were generally small, suggesting that other factors including biological and genetic, as well as other environmental stressors may be implicated in the development of psychopathology in children of veterans.

Our findings provide preliminary evidence that parenting may be a critical mechanism linking veteran depression and child psychopathology. Specifically, our results show that higher depression is linked to poorer parenting sense of competence in veterans and that poorer parenting sense of competence is associated with higher rates of child internalizing, externalizing, and attentional symptoms. These findings are based on cross-sectional data and thus do not inform the direction of these relationships. Our findings, however, provide preliminary evidence for the hypothesis that enhancing parenting sense of competence among veterans with depression may be a useful intervention target for reducing psychopathology in military-connected children; research examining the effect of this type of parenting intervention with military families is needed. Prior research indicates that parenting interventions can increase parental sense of competence [38]; thus parenting self-efficacy appears to be a modifiable target.

There are several reasons why parents who are struggling with depression may feel less capable of caring for their children. First, negative biases about the self that maintain depression are likely to carry over into self-perceptions of parenting. Additionally, symptoms of depression and resulting functional impairment may prevent veteran parents from interacting with children in ways that boost confidence. For example, parents with depression have been shown to exhibit less positive affect and more negative affect during observed interactions with their children [39]. Parents with depression may also find it more difficult to manage negative interactions with their children. For example, one study showed that depressed parents are more likely to use punitive disciplinary behaviors and are less likely to use some positive disciplinary strategies [40]. Understanding how depression is linked to poorer sense of competence in parenting would be valuable for identifying possible targets for intervention in this population.

Our study also revealed several ways in which veteran psychopathology and child psychopathology were not linked. Externalizing symptoms in children were not associated with any type of veteran psychopathology even though parenting sense of competence was linked to externalizing symptoms. This may suggest that for parents of children with externalizing problems, poor parenting sense of competence may stem from the challenges that come with dealing with the child’s difficulties rather than one’s own psychopathology; however we did not evaluate externalizing forms of veteran psychopathology in this study, so further research is needed. Moreover, veteran PTSD symptoms did not appear to be associated with child psychopathology or parenting sense of competence in this sample. These findings are inconsistent with previous research [13] and are particularly striking given that PTSD is often a primary focus in veteran samples. It is possible that differences may be due to our modified PCL, which combined different versions of the PCL that asked about different timeframes, though this is unlikely given the high internal reliability of our modified measure. Further research is needed to explore potential moderating factors that may explain these inconsistent results.

Several limitations should be taken into consideration when interpreting the results of this study. Our results are cross-sectional and relied exclusively on parental self-report. This means that directional interpretations cannot be made. It is possible that child psychopathology contributes to a diminished sense of parental competence, which in turns leads to parental depression. Moreover, it is possible that parental ratings of their children’s psychopathology could be influenced by their own mood states [30]. Longitudinal research is needed using multiple informants to explore causal links between parental psychopathology and child psychopathology in military families. Because we had no demographic information about the children, we were unable to determine whether child sex or age influenced the observed relationships. We also did not explore the co-parenting relationship between veterans and their partners or partner psychopathology. It is possible that veteran psychopathology may be associated with discord between the partners, which influences parenting and child psychopathology. It is also plausible that partner psychopathology may interact with veteran psychopathology in determining child outcomes. For example, having a partner with low psychopathology may serve as a protective buffer for children. Finally, we did not have a non-military control group; thus, it is impossible to establish the degree to which the observed results are driven by military experiences.

Despite these limitations, this study highlights the association between parental psychopathology and children’s mental health in military families. Moreover, the identification of the role of parenting competence in this relationship represents an important step towards understanding mechanisms that link veteran and child psychopathology. It is clear that military children represent an important high-risk group and that further quantitative and qualitative research is needed to understand the factors that enhance vulnerability and that can serve as potential targets for intervention.

## Summary

In this study, we evaluated the relationship between parent and child psychopathology in treatment-seeking veterans and explored parental sense of competence as a potential mediator of this relationship. We found high rates of child psychopathology in our sample, indicating that further attention is needed to screen for and address mental health concerns in this population. In particular, our findings suggest that veterans with higher rates of depression and anxious arousal may be more likely to have children with higher rates of internalizing symptoms and that veterans with higher rates of chronic tension may be more likely to have children with greater attentional problems. Parenting sense of competence significantly mediated the relationship between veteran depression and child psychopathology, suggesting that for veterans with depression and poor parenting sense of competence, interventions that aim to build parenting self-efficacy warrant further investigation as a strategy for enhancing child well being.
